# The protective effect of gentisic acid on rheumatoid arthritis via the RAF/ERK signaling pathway

**DOI:** 10.1186/s13018-022-03006-7

**Published:** 2022-02-20

**Authors:** Xiaojun Dong, Qi Zhang, Fujia Zeng, Mingxing Cai, Dou Ding

**Affiliations:** Traditional Chinese Medical Department of Zunyi Medical and Pharmaceutical College, Zunyi, 563006 Guizhou China

**Keywords:** Rheumatoid arthritis, Gentisic acid, FLS, RAF/ERK1/2

## Abstract

**Background:**

RAF and ERK pathways are known to be activated in human rheumatoid arthritis (RA) fibroblast-like synoviocytes (FLS), which play an important role in the pathogenesis and destruction of RA. Gentisic acid (GA) was a natural product derived from plants, which has been reported can attenuate pressure overload‐induced cardiac hypertrophy and fibrosis in mice through inhibition of the ERK1/2 pathway. Whether GA can inhibit the occurrence and development of RA through RAF/ERK signaling pathway has not been reported. The purpose of this study is to determine whether GA may have a certain therapeutic effect on RA-FLS.

**Method:**

Bovine type II collagen was used to establish a rat model of rheumatism. Enzyme-linked immunosorbent assay was used to detect inflammatory factors, anti-inflammatory mediators, and rheumatoid factor. Hematoxylin and eosin and TUNEL staining were used to detect the effect of GA on histochemical with rheumatoid arthritis. RAF, ERK, and p-ERK expressions in synovial tissue were measured by western blot and immunohistochemical. Besides, human rheumatoid arthritis fibroblast-like synoviocytes cell line MH7A was used to investigate the biological behavior influenced by GA. Apoptosis assay was performed to detect apoptosis of GA on MH7A cells. Transwell invasion assay was performed to detect the ability of cell migration.

**Result:**

The result showed that GA could reduce joint swelling and inflammation. At the same time, it can also promote the apoptosis of synovial cells and down-regulate the RAF/ERK pathway.

**Conclusion:**

GA may ameliorate inflammatory factors’ abnormality, synovial hyperplasia, and apoptosis of synovium via inhibiting the RAF/ERK signaling pathway.

## Introduction

Rheumatoid arthritis (RA) belongs to chronic autoimmune disease which has the main clinical manifestations of synovitis, cartilage damage, and symmetrical joint damage [1]. RA often occurs in small joints such as hands and feet, also involves other systems outside the joints, and even leads to joint deformities or loss of function [2]. The prevalence of rheumatoid arthritis is 5–10%, and there are regional differences [3]. The incidence of RA is determined by environmental factors, genetic background, environment-genetic interaction, and epigenetic modification [4]. The functional disability of RA patients will reduce their working ability and social participation, increase medical costs, and further aggravate the social burden [1]. Therefore, this is a major social problem that needs to be solved urgently.

Increasing studies implicated that MAPK pathway is activated in RA [5]. The MAPKs pathway, a critical signaling pathway in the pathogenesis of RA, is activated in FLSs when stimulated by pro-inflammatory cytokines, such as TNF-α and IL-1β [6, 7]. FLS plays an important role in joint balance, which exhibits independent growth, abnormal proliferation, and erosion characteristics. Besides, RA-FLS can also secrete a variety of inflammatory cytokines, which interact with local infiltrating inflammatory cells and jointly participate in the joint destruction process of RA [8, 9]. Rheumatoid factor (lgG, RF) is an antibody against the Fc region of IgG and has been used as a diagnostic marker for rheumatoid arthritis [10]. Inhibition of the MAPK (RAF/ERK1/2) signaling pathway can suppress the proliferation, migration, and invasion of RA-FLSs [11].

Touguxiang (TGX) is a famous Traditional Chinese medicinal herb used for the treatment of rheumatoid arthritis. TGX is the whole plant or root of Gaultheria leucocarpa Bl. var. crenulata (Kurz) T. Z. Hsu, which can cure rheumatoid arthritis by reducing the concentration of K^+^, DA, NE, and 5-HT in joint fluid, and the content of PGE2 or LTB4 in serum [12, 13]. Gentiolic acid (2,5-dihydroxybenzoic acid, GA) is the active ingredient of TGX, which can attenuate pressure overload‐induced cardiac hypertrophy and fibrosis in mice through inhibition of the ERK1/2 pathway [14, 15]. Although TGX has so many effects, it has not been reported whether it can regulate RA through the ERK pathway.

In this study, it discovered the therapeutic effect of GA on rheumatoid arthritis, explored its effect on the proliferation, apoptosis and aggressive behavior of FLSs in rheumatoid arthritis, and explored its potential mechanisms involved in the pathogenesis of rheumatoid arthritis.

## Materials and methods

### Animals and grouping

32 male Wistar rats (160 ± 10 g) were provided by the Chengdu Dashuo Experimental Animal Co. Ltd. The animal experiments were approved by the ethics committee of Traditional Chinese Medical Department of Zunyi Medical and Pharmaceutical College. The animal experiments meet the Canadian Council on Animal Care guidelines and National Academies Press (NAP) Guide for the Care and Use of Laboratory Animals. The rats eat and drink freely, the temperature is controlled at about 21 °C, the humidity is about 50%, the light is controlled for 12 h bright/12 h dark. Bovine type II collagen (BTII) (Chondrex, USA) was emulsified in complete Freund adjuvant (Sigma, USA) with the ratio of 1:1 to obtain a collagen emulsion. Except for the control group (*n* = 8), each rat was injected with 0.2 mL the mixture under the plantar fascia of the left hind foot to get the CIA rat model. The injection volume is 0.1 mL/mouse, and each animal is injected once [16, 17]. On the 7th day after the inflammation, the foot and plantar volume were measured. According to the different degree of plantar swelling, the rats were randomly divided into 3 groups, as the model group, GA low-dose (30 mg/kg), and high-dose group (60 mg/kg). On the 7th–28th day after inflammation, the rats were given continuous intragastric administration once a day, and the normal group and the model group were given equal volumes of normal saline. On the 28th day, the rats were the animals were injected 1% sodium pentobarbital (50 mg/kg) and sacrificed by carbon dioxide inhalation to obtain tissue and blood.

### Enzyme-linked immunosorbent assay (ELISA)

After resting at 4 °C for 10 min, rat blood was centrifuged at 1000 rpm for 10 min to obtain serum. The rat serum rheumatoid factor (RF), interferon-γ (IFN-γ), interleukin-1β(IL-1β), interleukin-4(IL-4), interleukin-10 (IL-10), and tumor necrosis factor-α (TNF-α) were detected by ELISA kits (Jianglai Biological Co., Ltd.,Shanghai, China).

### H&E staining and immunohistochemistry (IHC) staining

Synovial tissue of the joint from rat was fixed in 4% paraformaldehyde for 24 h. The tissues were dehydrated with ethanol and xylene, fixed in paraffin and sectioned (4 μm) for staining. The synovial tissue of the joint sections was stained with hematoxylin and eosin (H&E) and the images were acquired under a light microscope equipped with 10× or 40× objective lens. For IHC staining, the paraffin sections were deparaffinized with xylene and 3% hydrogen peroxide for antigen retrieval at room temperature for 10 min. Then, the sections were incubated with primary antibodies against RAF(1:500; ab125212; Abcam, CA, USA) at 4 °C overnight, followed by incubation with the appropriate amount of HRP goat anti-rabbit IgG (1:5000; ab205718; Abcam, CA, USA) for 30 min at 37 °C. Next, the reaction was visualized using DAB (Boster, Wuhan, China). Five visual fields were randomly selected and assessed for immunoreactive areas at ×200 magnification using BA400Digital microscope (Motic, Xiamen, China). The optical density of image was analysed by Image-Pro Plus software (Media Cybernetics, Inc., Rockville, MD, USA).

### Terminal deoxynucleotidyl transferase dUTP nick end labeling (TUNEL) staining

The tissue from synovium of joint was incubated with proteinase K at 37 °C for 30 min in the dark to make the cell model permeable. 100 μL 1 × Equilibration Buffer was used to fix it for 10–30 min. Then, aspirate the supernatant and incubate it with Alexa Flour 488-dUTP Labeling Mix at 37 °C for 1 h, wash away the background with PBS, seal the cell slide with DAPI-containing mounting tablets, and observe and collect under a laser confocal microscope.

### Western blot assay

Proteins were extracted from MH7A cells, ankle joint and knee joint tissues using RIPA lysis buffer (BeyoTime Biotech, Shanghai, China) and quantified by a BCA Protein Assay Kit (BeyoTime, China). A total of 20 μg protein sample was separated by 8% SDS-PAGE and blotted onto PVDF membrane. After blocked with 5% non-fat milk in TBST for 1 h at room temperature, the membranes were then incubated with appropriate primary antibodies at 4 °C overnight, followed by probed with secondary antibody (Abcam, USA, ab205718) conjugated to horseradish peroxidase. The protein signals were visualized using an ECL detection system. The primary antibodies including RAF (Abcam, USA, ab170099), ERK1/2 (Abcam, USA; ab184699), p-ERK1/2 (Abcam, USA, ab76299).

### Cell culture and treatment

Human rheumatoid arthritis fibroblast-like synoviocyte cell line MH7A was purchased from Procell Life Science & Technology Co., Ltd. (Wuhan, China). Cells were divided into 4 groups. The blank control cells were cultured as normal. The concentration of GA treatment group is 10 μg/mL. RAF overexpression group, the plasmid concentration of pcDNA3.1RAF (Sangon Biotech (Shanghai) Co., Ltd.) was 100 μg/mL. The fourth is GA and pcDNA3.1RAF combined treatment group. MH7A cells were cultured in DMEM/high glucose medium (Hyclone, USA) supplemented with 10% fetal bovine serum (FBS, Gibco, USA) and 1% glutamine (Sigma, USA) and incubated at 37 °C under 5% CO_2_.

### Cell proliferation assay

Cell Counting Kit-8 (CCK-8) (Dojindo, Japan) assay was used to measure cell proliferation. MH7A cells in logarithmic growth phase were seeded in a 96-well plate (6 × 10^3^/well). After the cells were attached to the wall, the cells were incubated with GA (10 μg/mL) or pcDNA3.1 RAF plasmid (100 μg/mL) for 48 h, and then the cells were incubated with 10% CCK-8 solution for 1 h at 37 °C under 5% CO2. The absorbance value was measured using a microplate reader under 450 nm.

### Apoptosis assay

Annexin V-APC/PI staining and flow cytometry (FCM) were performed to detect apoptosis of MH7A cells. Cells were double-labeled with Annexin V-APC/PI (Abnova, China) in the dark at room temperature for 15 min. Then, the FACSCalibur flow cytometer of BD Biosciences in the United States was used to analyze the effect of GA on cell apoptosis.

### Transwell invasion assay

0.25% trypsin-EDTA solution with a cell density of 2 × 10^5^/ml was used for transwell invasion assay. 50 μl of Matrigel (BD Biosciences, Franklin Lakes, NJ, USA) was added on the membrane of the upper chamber and solidified at 37 °C for 30 min. 600 μl of complete medium containing 10% fetal bovine serum (FBS; Gibco, Grand Island, NY, USA) was added to a 24-well plate, and 100 μl of cell suspension was seeded on the membrane and incubated at 37 °C in an atmosphere of humidified air and 5% CO_2_ incubator. Transwells were collected 24 h after incubation, fixed in − 20℃ cold methanol for 30 min, and then stained with 0.5% crystal violet (Solarbio, Beijing, China) at room temperature for 20 min. Invasive cells were observed under an optical microscope (Olympus, Tokyo, Japan). Assays were independently repeated three times.

### Statistical analysis

One-way ANOVA was performed for statistical analysis (SPSS 19.0) Values were presented as the means ± standard deviation (SD). Differences among multiple groups were compared by one-way analysis of variance (ANOVA) with Dunnett’s post-tests The differences were considered statistically significant at *P* < 0.05 and *P* < 0.01.

## Results

### The therapeutic effect of GA on rheumatoid arthritis

**Fig. 1:**
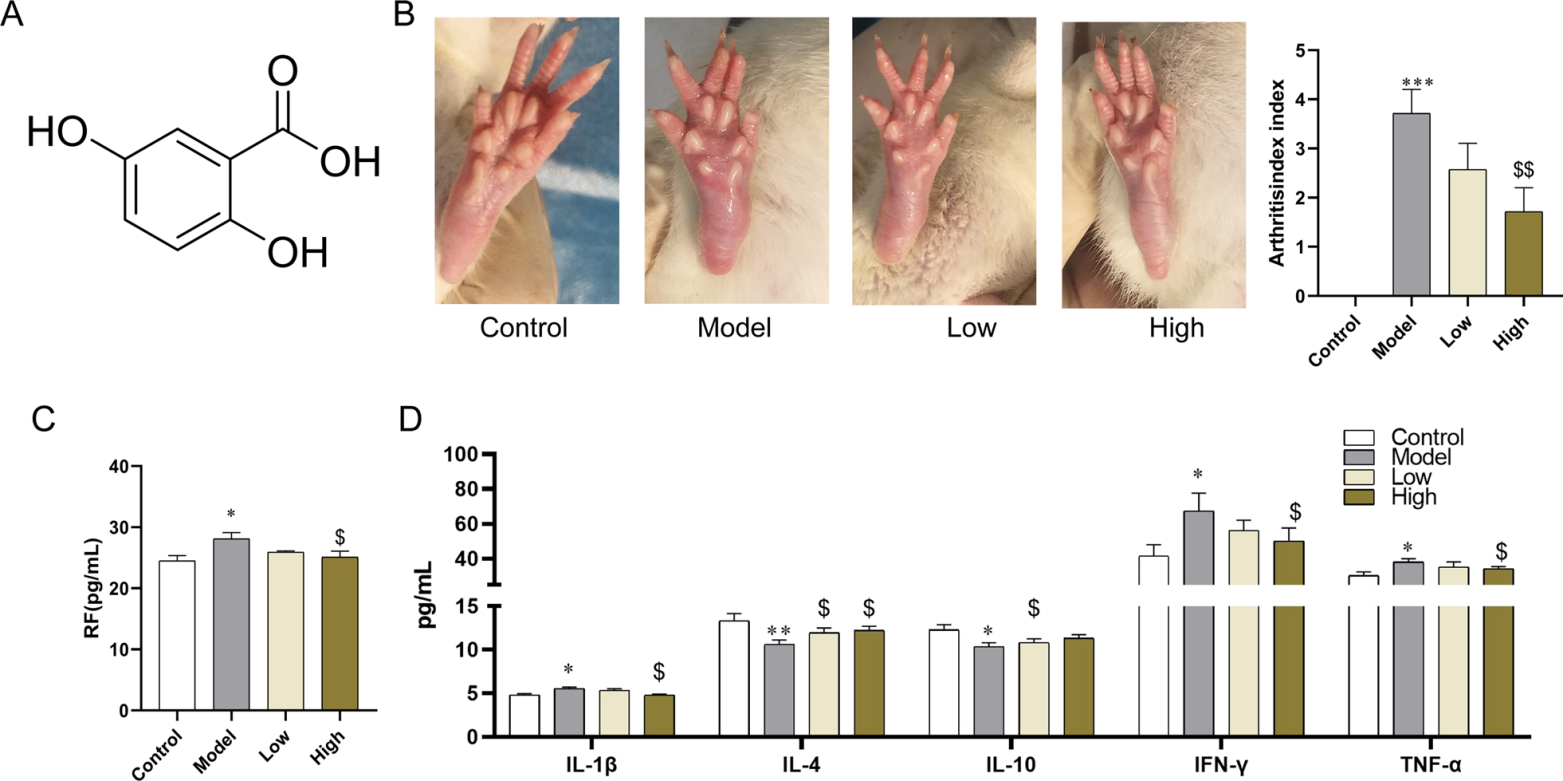
The therapeutic effect of GA on rheumatoid arthritis. **A** 2D structure of gentisic acid. **B** Representative picture of joint swelling in CIA rats. **C** The effect of GA on serum RF levels. **D** The effect of GA on serum IFN-γ IL-1β, TNF-α, IL-4, and IL-10 levels. Arthritis index: 0 means no redness and swelling; 1 means little toe joints are slightly swollen and red; 2 means more than one joint is swollen and red; 3 means toe joints and feet are swollen; 4 means ankle joints and feet below the ankle joints The paws are swollen. *P < 0.05, **P < 0.01, and ***P < 0.001 compared with the control group. ^$^*P* < 0.05, ^$$^*P* < 0.01, and ^$$$^*P* < 0.001 compared with model group

GA is a quinonoid phenolic acid synthesized in plants (Fig. 1A). To study the therapeutic effects of GA on RA, the CIA rat model was chosen to be the subject. As shown in Fig. 1B, there was hardly any swelling in the control group. However, joint swelling was observed clearly in the model group. Compared with the model group, the level of joint swelling was alleviated in 30 mg/kg and 60 mg/kg GA treatment rats. To prove GA can restore abnormal changes of inflammatory mediators, anti-inflammatory mediators, and rheumatoid factors in serum were detected. As shown in Fig. 1C, D, the levels of INFc, IL-1β, TNF-α, and rheumatoid factor (RF) in the model group was significantly higher than those in the control group, while the levels of the above-mentioned inflammatory mediators and RF have significantly decreased following GA treatment. The levels of IL-4 and IL-10 in the model group were significantly lower than those in the control group. However, compared with the model group, they were significantly increased in the 30 mg/kg and 60 mg/kg GA treatment groups (Fig. 1D). These results indicated that GA can treat RA by reducing inflammation.

### The effect of GA on histochemical with rheumatoid arthritis

**Fig. 2:**
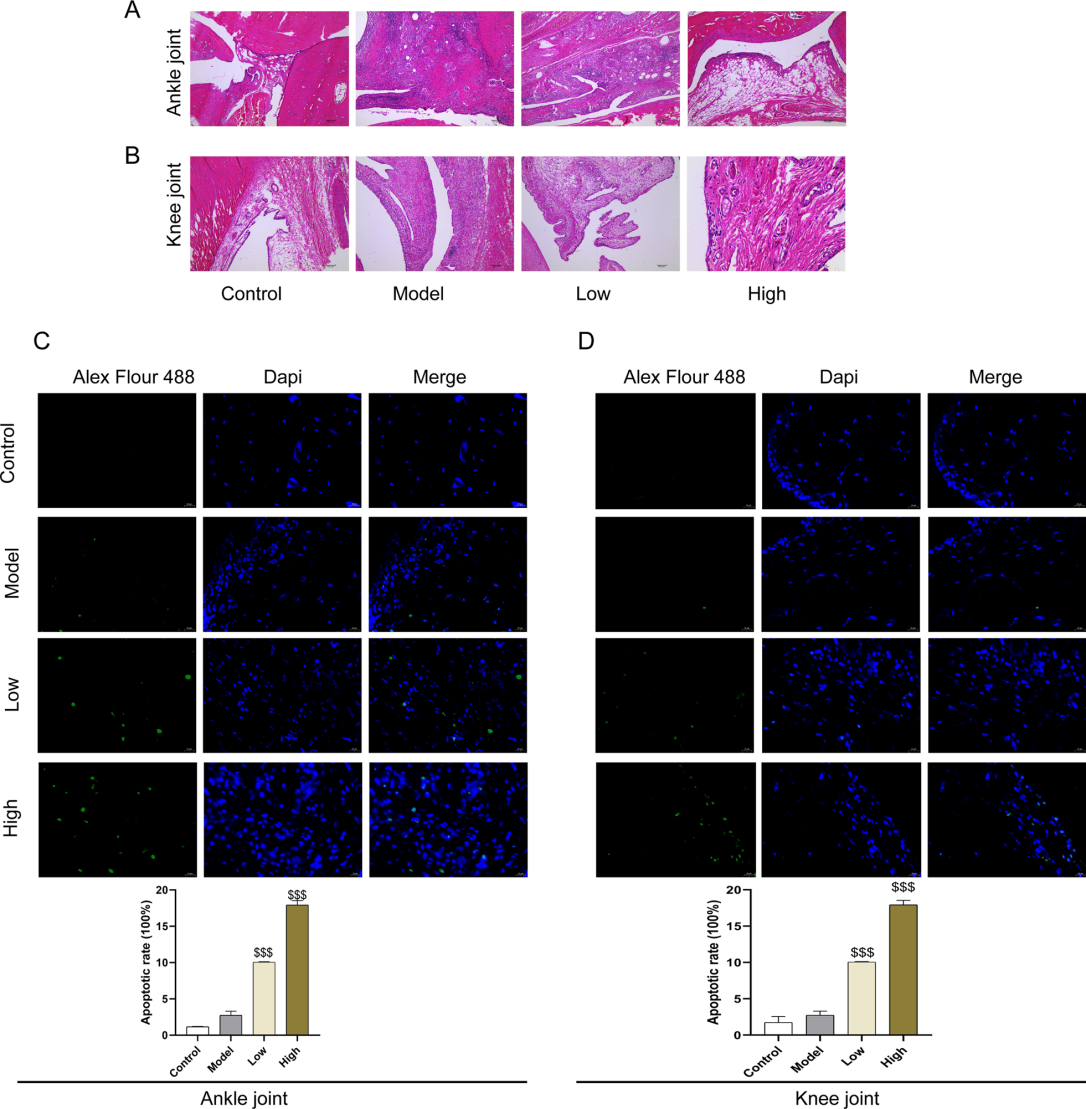
The effect of GA on histochemical with rheumatoid arthritis. **A** Images of ankle synovial tissue following hematoxylineosin (H&E) staining. **B** Images of knee synovial tissue following hematoxylineosin (H&E) staining. **C** Images of ankle synovial tissue following TUNEL staining. **D** Images of knee synovial tissue following TUNEL staining. *P < 0.05, **P < 0.01, and ***P < 0.001 compared with the control group. ^$^*P* < 0.05, ^$$^*P* < 0.01, and ^$$$^*P* < 0.001 compared with model group

To determine whether GA restored pathological changes in Synovial Tissue, HE staining was performed on synovial tissue of the ankle and knee joints. As shown in Fig. 2A, the synovial structure of the ankle joint in the control group was clear and complete, with connective tissue composed of more fat cells, a few fibroblasts, macrophages, and a few blood vessels. The synovial tissue had no inflammatory cell infiltration and fibrous tissue hyperplasia. In the model group, the thickness of the synovial lining of the ankle joint was significantly thicker, and more inflammatory cells were infiltrated, including lymphocytes, plasma cells, and a small number of macrophages. Moreover, in the lower layer of the synovial lining, a large number of the proliferation of fibrous tissues can be seen, and the proliferation of fibroblasts with an oblong nucleus can be seen. The above-mentioned adverse reactions were alleviated under GA treatment. As shown in Fig. 2B, the synovial structure of the knee joint in the control group was clear and complete. The lining layer was composed of a single layer or a double layer of synovial cells, and no inflammatory cell infiltration and fibrous tissue hyperplasia. Synovial cells in the model group proliferated significantly, and the lower layer was multi-fibered, and fibroblasts with oval nuclei. The lower layer is infiltrated with multiple inflammatory cells, including lymphocytes, neutrophils, and macrophages. The adverse reactions caused by BTII under the treatment of low and high doses of GA are close to those of the control group. Apoptotic cells were detected by TUNEL staining (Fig. 2C, D), and the positive cells were stained by Alexa Flour 488-dUTP Labeling Mix as green. The control group and model was no green fluorescence, while the GA treatment group had more green fluorescent cells than the model group. These results indicated that GA can improve synovial hyperplasia and inhibit apoptosis of synovial tissues in CIA rats.

### GA inhibited the activation of RAF/ERK1/2 pathway in synovial tissue

**Fig. 3:**
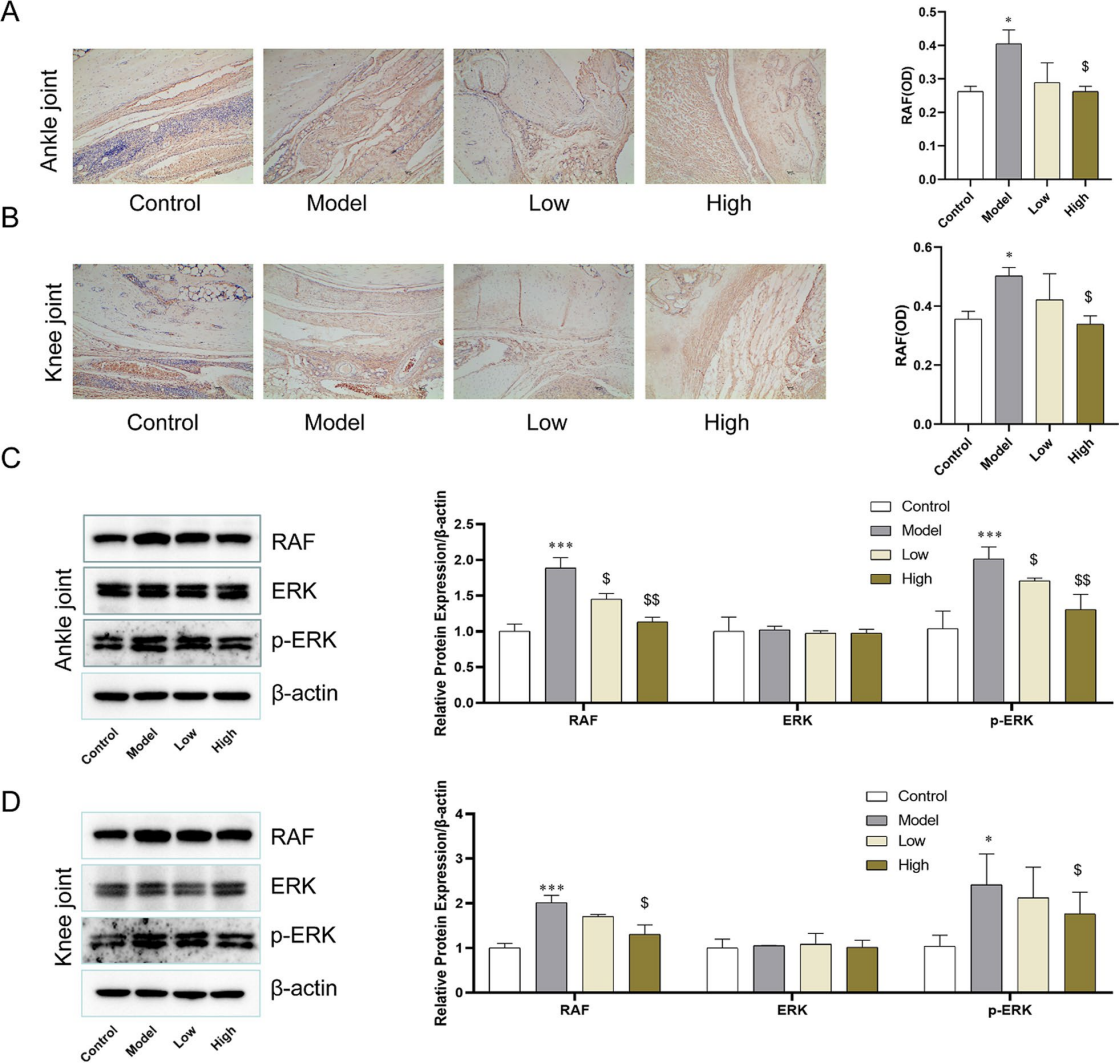
RAF, p-ERK1/2, and ERK1/2 protein levels in synovial tissue of ankle joint and knee joint. **A** Immunohistochemical analysis of RAF in ankle synovial. **B** Immunohistochemical analysis of RAF in knee synovial. **C** The effect of GA on the expression of RAF, ERK, and p-ERK in ankle synovial. **D** The effect of GA on the expression of RAF, ERK, and p-ERK in knee synovial. *P < 0.05, **P < 0.01, and ***P < 0.001 compared with the control group. ^$^*P* < 0.05, ^$$^*P* < 0.01, and ^$$$^*P* < 0.001 compared with model group

To explore the regulatory mechanism of GA inhibits RA, the RAF/ERK signaling pathway was detected by immunohistochemistry and western blot assays. Compared with the control group, the expression of RAF was significantly increased in the model group. However, the levels of RAF in ankle and knee joints induced by Bovine type II collagen were reversed by GA especially within high dosage (Fig. 3A, B). At the same time, Phosphorylation of ERK1/2 was significantly increased in the model group, almost twofold compared to the control group, while it was significantly decreased in each dose of the GA treatment group (Fig. 3C, D). This result indicated that the RAF/ERK1/2 signaling pathway was activated in CIA rats, and can be reversed by the high concentration of GA.

### The biological effects of RAF/ERK1/2 pathway on MH7A cells

**Fig. 4:**
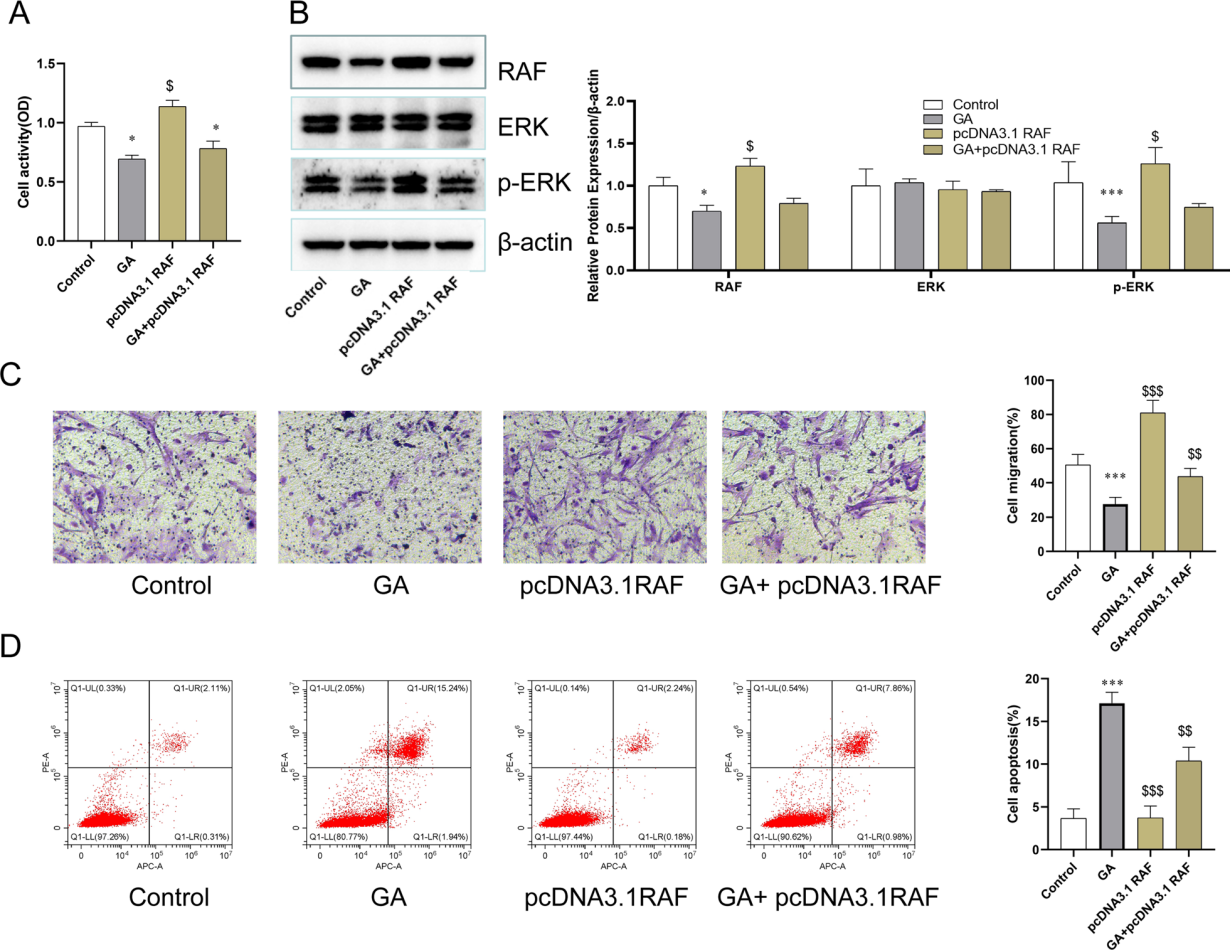
GA inhibited the activiation of RAF/ERK1/2 pathway to influence the biological behavior in MH7A Cells. **A** Cell viability of MH7A cells. **B** The effect of GA on the expression of RAF, ERK, and p-ERK in MH7A cells. **C** Transwell assay of on MH7A cells. **D** Apoptotic rate of MH7A cells. *P < 0.05, **P < 0.01, and ***P < 0.001 compared with the control group. ^$^*P* < 0.05, ^$$^*P* < 0.01, and ^$$$^*P* < 0.001 compared with model group

To further investigate the effect of GA on MH7A cells damage is through activation of the RAF/ERK1/2 pathway, MH7A cells were transfected with pcDNA3.1 RAF plasmid and detected by, CCK8, Transwell invasion, and FCM assays. The optimal concentration of 10 μg/mL was obtained through preliminary experiments. Compared with the control group, overexpressed RAF by pcDNA3.1RAF has the effect of promotes cell proliferation and inhibits apoptosis. GA can reduce the high expression of RAF, which can further affect the increase in cell proliferation and the decrease in apoptosis caused by overexpression of RAF (Fig. 4A, D). As shown in Fig. 4C, GA can inhibit cell invasion, while overexpressed RF has the opposite effect. To further testify the signal transduction pathway of GA, a western blot assay was performed to detect the expression of RAF/ERK in MH7A cells (Fig. 4B). Compared with the control group, pcDNA3.1RAF can significantly overexpress RAF. GA can inhibit the expression of protein RAF and p-ERK, while pcDNA3.1RAF can promote the expression of RAF and even p-ERK, so the activation of RAF can further activate ERK (Fig. 4C). These data indicated that GA could inhibit cell viability and promote apoptosis in MH7A cells via RAF/ERK1/2 pathway.

## Discussion

With the continuous improvement of living standards of Chinese residents and the increasing pressure of work and life, the number of patients with RA is increasing.

The Miao, Bai, and Yi ethnic minorities in my country have experience in applying TGX has significant effects in the treatment of muscle soreness, rheumatism, lumbar spine hyperostosis, ankle sprain, and rheumatic arthralgia [18]. In this study, we demonstrated the effect of gentisic acid on CIA model rats through RAF/ERK pathway and verified it again in RA-FLS cells for the first time. We confirmed that GA could alleviate systemic inflammation, synovial hyperplasia, and MH7A cell proliferation. We also find that GA can influence the invasiveness of RA-FLS.

It's well known, serum inflammatory factors and rheumatoid factors are important characteristicss of rheumatoid arthritis. Many studies have demonstrated that TNF-α, IFN-γ, and IL-1β in the peripheral blood of RA of patients are significantly increased [19, 20]. The reduction of a variety of anti-inflammatory factors in serum accelerates the pathological process of the disease, including IL-4 and IL-10 [21, 22]. The increase of RF in the model group proved that the CIA model prepared during the experiment was successful, and could be reversed high-dose of GA. The results of this study also showed that GA can inhibit TNF-α, IFN-γ, and IL-1β caused by BTII, and it can also inhibit the down-regulation of IL-4 and IL-10 caused by BTII. The therapeutic effect of the high-dose group is particularly obvious, suggesting that the therapeutic effect of GA on CIA rats may be through regulating inflammatory factors and RF directly. Regarding H&E and TUNEL staining experiments, we found that the inflammation and synovial hyperplasia of synovial tissue in CIA rats were improved after GA was administered. In addition, the administration of GA also promoted synovial cell apoptosis. The above results indicated that GA has the effect of inhibiting the proliferation of rheumatoid arthritis synovial cells, and the therapeutic effect of the drug may be achieved by promoting cell apoptosis.

RAF and ERK are the two key proteins in the MAPK signaling pathway, and the MAPKs pathway is the critical signaling pathway in the pathogenesis of RA [23, 24]. The Muller-Ladners' study showed that when MAPKs were activating, the proliferation ability of RA-FLS became strong [25]. Zhangs' experiment proved that overexpression of Spry2 could attenuate the proliferation of Raf/ERK pathway and RA FLSs [11, 26]. In this research, the results of immunohistochemistry and western blot showed that RAF and ERK were highly expressed in rat joint synovium tissues, and they could be activated by COII. At the same time, GA had a significant inhibitory effect on the expression of Raf and ERK proteins. Therefore, GA seems to have an inhibitory effect on the expression of Raf and ERK proteins, indicating that the GA may inhibit the RAF/ERK1/2 pathway to achieve the therapeutic effect of RA.

Additionally, GA has an inhibitory effect on RA-FLS through the RAF/ERK pathway. The results showed that GA could inhibit the biological behavior of MH7A, such as proliferation and invasion. The result of Flow cytometry experiments showed that the activation of RAF has the effect of inhibiting cell apoptosis. The result of western blot showed that compared with the control group, GA could inhibit the expression of protein RAF and p-ERK. While, pcDNA3.1RAF can promote RAF, and the activation of the RAF can further activate ERK. These experimental results show that RAF can activate ERK and affect the proliferation of RA-FLS, and RAF may be a potential target for RA treatment.

## Conclusions

GA significantly inhibits joint inflammation by regulating Raf/ERK signals, suggesting that GA-based immunomodulation strategies may have therapeutic potential in the treatment of RA. At the same time, GA can also inhibit the proliferation of RA-FLS and promote apoptosis to achieve the purpose of treating RA through the Raf/ERK pathway.

## Data Availability

Not applicable.

## Abbreviations

GA: Gentisic acid
RA: Rheumatoid arthritis
FLS: Fibroblast-like synoviocytesl
TGX: Touguxiang
BTII: Bovine type II collagen
